# Cyclitols: From Basic Understanding to Their Association with Neurodegeneration

**DOI:** 10.3390/nu15092029

**Published:** 2023-04-23

**Authors:** Maria Derkaczew, Piotr Martyniuk, Adam Osowski, Joanna Wojtkiewicz

**Affiliations:** 1 Department of Human Physiology and Pathophysiology, School of Medicine, Collegium Medicum, University of Warmia and Mazury, 10-082 Olsztyn, Poland; p.martyniuk@o2.pl (P.M.);; 2 Students’ Scientific Club of Pathophysiologists, Department of Human Physiology and Pathophysiology, School of Medicine, University of Warmia and Mazury, 10-082 Olsztyn, Poland

**Keywords:** myo-inositol, d-pinitol, inositol phosphates, neurodegenerative disorders, Alzheimer’s disease, Parkinson’s disease, Huntington’s disease, spinocerebellar ataxias

## Abstract

One of the most common cyclitols found in eukaryotic cells—Myo-inositol (MI) and its derivatives play a key role in many cellular processes such as ion channel physiology, signal transduction, phosphate storage, cell wall formation, membrane biogenesis and osmoregulation. The aim of this paper is to characterize the possibility of neurodegenerative disorders treatment using MI and the research of other therapeutic methods linked to MI’s derivatives. Based on the reviewed literature the researchers focus on the most common neurodegenerative diseases such as Alzheimer’s disease, Parkinson’s disease, Huntington’s disease and Spinocerebellar ataxias, but there are also works describing other seldom encountered diseases. The use of MI, d-pinitol and other methods altering MI’s metabolism, although research on this topic has been conducted for years, still needs much closer examination. The dietary supplementation of MI shows a promising effect on the treatment of neurodegenerative disorders and can be of great help in alleviating the accompanying depressive symptoms.

## 1. Introduction

Over the centuries, plants as natural products were used to treat a multitude of diseases. These organic “drugs” contain numerous chemical particles. One of them—myo-inositol (MI) and its derivatives—play an influential role in a great number of cellular processes such as ion channel physiology, phosphate storage, signal transduction, cell wall formation, membrane biogenesis and osmoregulation as it is a forerunner of many secondary messengers in eukaryotic cells [1,2].

MI is a pivotal and fundamental element of Inositol Phosphates (IPs) and Phosphatidylinositols (PIs or PtdIns) and their various phosphates, called phosphatidylinositol phosphate lipids (PIPs). Their interconversions are conducted by crucial enzymes, which dysfunction can lead to severe abnormalities, disorders and illnesses.

The aim of this paper is to present and analyze the documented data about cyclitols’ association with pathological processes such as neurodegenerative diseases. Furthermore, cyclitols themselves may be worth considering as therapeutic agents in the treatment of numerous medical conditions. Low toxicity, possible use in pregnancy, good drug tolerance, widespread presence, and easy availability speak for use of these compounds in medicine [3]. The first part of this paper presents general information concerning the sugar alcohols, inositol and its derivatives such as PIs with the detailed characterization of metabolic pathways in this particular group and selected genetic mutations concerning PIs found in the literature; then the next part of the review focuses on the exact characteristic of IPs, to finally move on to discuss the neurodegenerative diseases connected with disorders in MI metabolic pathways and its possible usage in the treatment of these diseases as well as other innovative treatment methods.

## 2. Sugar Alcohols

Cyclitols are monocyclic saturated hydrocarbons containing at least three hydroxyl groups, each attached to a different ring carbon atom. The other names of this group of organic compounds are sugar alcohols or polyols. Cyclitols and their derivatives are tiny organic molecules with low toxicity and neutral charge, which makes them osmoprotectants. They are formed in plants to help them survive extreme osmotic stress. That is one of the various and numerous functions that cyclitols can perform. MI, being one of the sugar alcohols and most common cyclitols in eukaryotic cells, serves as a key component of structural lipids such as IPs, PIs and PIPs [4] (Table 1).

## 3. Inositol Characteristics

Inositol is a cyclic carbohydrate with six hydroxyl groups attached to each ring carbon. MI is one of nine inositol isomers as each hydroxyl group of the cyclohexane ring can be placed in one of two possible orientations—axial or equatorial [5]. The other eight are d-chiro-, scyllo, muco-, *cis*-, neo-, epi-, allo- and l-chiro-inositol. d-chiro- and l-chiro-inositol are two enantiomers, which means they are mirror images of each other. MI undergoes many biochemical and metabolic changes to form different stereoisomers and transforms into other more complex molecules such as IPs, PIs, PIPs, glycosyl-phosphatidylinositols (GPIs) and inositol-phosphoglycans (IPGs) (2). MI or *cis*-1,2,3,5-*trans*-4,6-cyclohexanehexol is the oldest known inositol and at the same time the most abundant one in nature [6]. It was first isolated in 1850 by Scherer from muscle extracts and named after that tissue with prefix “myo” from Greek. Other isomers that occur naturally in nature are: d-chiro-, l-chiro, scyllo-, muco- and neo-inositol [5]. Inositol is commonly called vitamin B8, which is not entirely precise due to the fact that this compound is also synthesized endogenously [7]. The functions of MI in the human body include the regulation of ion channel permeability, metabolic homeostasis, export and the translation of mRNA, stress reaction, cytoskeleton remodeling, apoptosis, fetus development, the development and function of nerves, reproductive functions, osteogenesis and metabolism of glucose and cholesterol [2,8].

## 4. Sources and Metabolism of MI

MI is primarily supplied with food. The highest amounts of MI can be found in plant-based products, such as grains, nuts and fruits. It is the main source of phosphorus in plant cells and is responsible for its storage [9]. MI occurs in food in its free form, but mainly in the form of phytic acid (IP_6_) which is known for its both upsides and downsides. It has long been known that IP_6_ binds to bivalent cations such as Mg^2+^, Zn^2+^, Ca^2+^, Fe^2+^, and Mn^2+^ and forms phytases that cannot be broken down due to the lack of a predestined enzyme, contributing to the long term to deficiency of these minerals [10]. Newer studies indicated that approximately 50% of IP_6_ can be degraded in the stomach and small intestine when the diet contains plant food phytases [11]. IP_6_ is also digested by bacterial phytases and phosphatases [12]. On the other hand, IP_6_ is a natural antioxidant. The thermal or mechanical treatment of the food such as cooking, soaking, baking and general processing significantly reduces the IP6 content [13]. It has been estimated that an average mixed Western diet can provide about 1 g of total inositol per day [14].

There are two types of transporters which are enabling MI cell uptake: sodium ion coupled (SMIT1/SMIT2) and proton coupled (HMIT1) [15,16,17]. HMIT1 expression is most significant in the brain [18].

**Figure 1:**
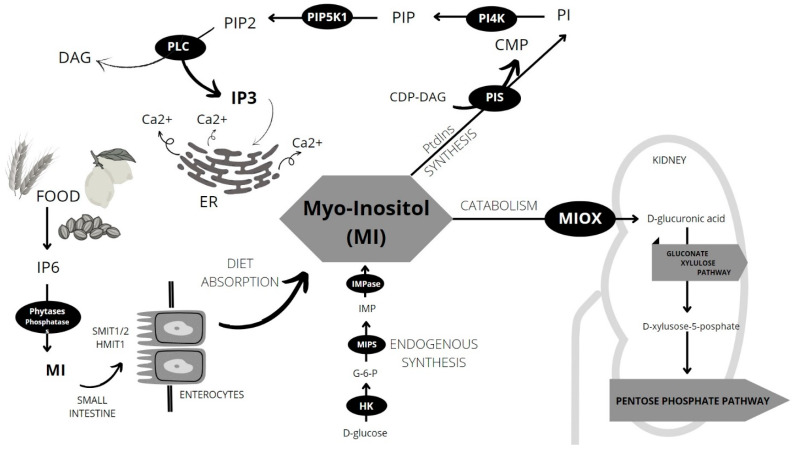
Metabolism of MI [IP6—phytic acid; HK—Hexokinase; G-6-P—Glucose-6-Phosphate; MIPS—Myo-inositol Phosphate Synthase; IMP—inositol Monophosphate; IMPase—Inositol Monophosphatase; MIOX—Myo-inositol Oxygenase; CDP-DAG—Cytidine Diphosphate Diacylglycerol; PIS—Phosphatidylinositol Synthase; CMP—Cytidine Monophosphate; PI—Phosphatidylinositol; PI4K—Phosphatidylinositol 4-Kinase; PIP—Phosphatidylinositol Phosphate; PIP5K1—Phosphatidylinositol Phosphate 5-Kinase 1; PIP2—Phosphatidylinositol Biphosphate; PLC—Phospholipase C; DAG—Diacyloglicerol; IP3—Inositol Triphosphate; ER—Endoplasmic Reticulum].

MI can be also endogenously produced in the human kidney and liver from glucose-6-phosphate in just two reactions. The first one is catalyzed by MI-1-phosphate synthase (MIPS) and the second one by inositol monophosphatase-1 (IMPase) (Figure 1) [19,20,21]. To date, no dietary requirements regarding the amount of inositol daily intake have been established for humans. It is probably hampered due to the endogenous production of MI from glucose and differences in MI levels in individual organs and compartments [2].

The kidney is the main organ of MI metabolism. Here, endogenous biosynthesis as well as catabolism takes place. MI is oxygenated to form d-glucuronic acid which can enter the gluconate xylulose pathway and be converted to d-xylulose-5-phosphate—one of the key components of the pentose phosphate pathway [8,22]. The pentose phosphate pathway is one of the most important processes in the human body. It allows the generation of energy and the maintenance of homeostasis, also being the principal source of nicotinamide adenine dinucleotide (NADPH), pentoses and ribose-5-phosphate—nucleotides synthesis precursor [23,24].

MI is a forerunner of many secondary messengers which play a key role in various intracellular processes. The most important derivatives are a family of phosphatidylinositols and inositol phosphates, especially inositol triphosphate (IP_3_) [8].

## 5. Inositol Phosphates

Inositol phosphates (IP) are derivatives of MI. Among the IPs we can distinguish: IP, IP2, IP3, IP4, IP5 and IP6—also known as phytic acid. The level of IP6 is highest in nerve cells and is relatively constant regardless of the supply of IP6 in the diet [2]. IPs play a key role in the biochemistry and metabolism of cells. IP3 is an important secondary messenger, resulting from the breakdown of PIP2 by phospholipase C along with diacylglycerol (DAG). DAG remains bound to the cell intracellular membrane, while soluble IP3 is released into the cytoplasm and binds to the inositol 1,4,5-triphosphate receptor (IP3R). IP3R activation enables intracellular calcium (Ca^2+^) signaling as it leads to the opening of Ca^2+^ ion channels located on the endoplasmic reticulum (ER) and the release of calcium ions into the cytoplasm.

There are three types of these receptors: IP3R 1, IP3R 2 and IP3R 3 [25]. IP3R 1 is the most common one expressed in neurons of all three types. Two receptors are responsible for the release of calcium form the ER: IP3R and the ryanodine receptor (RyR). RyR is the primary one in the striated muscle cells, while IP3Rs can be detected in all cell types. The function of these channels contributes to regulating various physiological functions such as muscle contraction, hormone secretion, reproduction, gene expression, cell life cycle, apoptosis, learning, memory and behavior [26].

Calcium release from the ER can be induced both by inositol (Inositol Induced Calcium Release; IICR) or calcium itself (Calcium Induced Calcium Release; CICR). While IP3Rs are solely responsible for IICR, CICR is responsible for the release of calcium from both IP3R and RyR, but with a significant advantage of RyR in this process [27]. Changes in the regulation of IP3Rs can lead to various abnormalities. Deregulation of these receptors disturbs intracellular homeostasis lowering the concentration of Ca^2+^ in the cytoplasm, changing the amplitude and frequency of calcium signaling and ultimately can even lead to cell death [25,28]. It was proven that improper function of these channels can lead to many neurological disorders including migraines, Alzheimer’s disease (AD), Huntington’s disease (HD) and spinocerebellar ataxias [25,26]. It is generally accepted that IP3Rs localize on the ER, but where else they may exist within the cell is under discussion. For the time being, it is known that these receptors may also appear on the inner nuclear membrane and within the plasma membrane. In neuronal cells presence of IP3R on the plasma membrane has so far been detected only in olfactory receptor neurons [29].

## 6. Inositol’s Unproper Metabolism Impact on Neurodegenerative Process

In the brain, constant changes in inositol levels in extracellular and intracellular compartments regulate the activity of neuronal and glial cells [30]. MI and scyllo-inositol (SI) are the most common forms of inositol found in the brain. MI is found in large amounts in the glial cells and acts as an osmolyte. The concentrations of MI and SI are several times higher than the values found in the blood serum and are on average 100 times higher [31,32]. In the nervous system, the highest concentration of inositol is in the brain—6 mM. Then, MI occurs in cerebrospinal fluid (0,2 mM) and plasma of nervous cells (0.03 mM) [3,33,34]. SI is found in relatively high concentrations in the human brain than in other tissues, although it has a 12 times lower concentration than mI [35,36]. In nervous system diseases, we notice the occurrence of unproper MI’s metabolism to phosphates and its secondary accumulation. MI has been found to be a marker of gliosis in demyelinating lesions. Elevated levels of MI have also been documented in glioblastomas. MI is also involved in the phosphatidylinositol secondary messenger system, which is associated with depression (91). Disorders in MI’s metabolism and distribution have been found in many neurodegenerative diseases.

### 6.1. Alzheimer’s Disease

**Figure 2:**
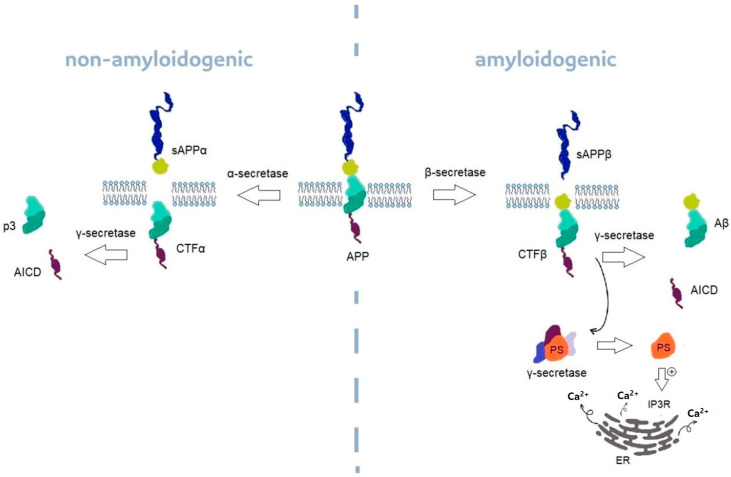
The amyloidogenic and non-amyloidogenic cleavage of amyloid precursor protein and the linkage between γ-secretase and IP3R [APP—amyloid precursor protein; AICD—APP intracellular fragment; CFT—APP C-terminal fragment].

Alzheimer’s Disease (AD) is a chronic, progressive, neurodegenerative disease of the brain that causes nerve cell atrophy. AD is characterized by the occurrence of progressive dementia and at the same time is the most common cause of dementia among the elderly [37]. In the course of AD, we can distinguish symptoms such as memory loss and the progressive loss of cognitive functions [38]. Common neuropathological features of AD are neuronal loss, the aggregation of insoluble forms of β-amyloid (Aβ) forming plaques in extracellular spaces and microtubule τ protein hyperphosphorylation in the form of neurofibrillary tangles (NFTs) located in neuronal cells. Changes are usually located firstly in the cortex of frontal and temporal lobes and then slowly spread to the other parts of the brain tissue [39]. We can distinguish the sporadic form of AD (Sporadic Alzheimer’s Disease—SAD) and the familial form (Familial Alzheimer’s disease—FAD). FAD occurs much less frequently in about 5% of AD patients [40]. SAD tends to occur in the elderly and is the most common form of AD, while FAD will appear more frequently in middle age and is associated with mutations in the genes encoding the presenilin 1 and 2 proteins (PS 1,2) and the amyloid precursor protein (APP) [25,38,41,42,43]. APP is normally transformed through several reactions of proteolysis into two types of Aβ—Aβ40 and Aβ42. γ-secretase is one of the enzymes catalyzing these reactions and will be mentioned later in this paper, as a possible therapeutic target in AD treatment. The possible linkage between γ-secretase and inositol metabolism is the fact that PS1 is the main catalytic core of γ-secretase and PS1 is known to boost the activity of IP3R. These reactions and the cleavage of APP can be seen on Figure 2 [2,44,45].

**Figure 3:**
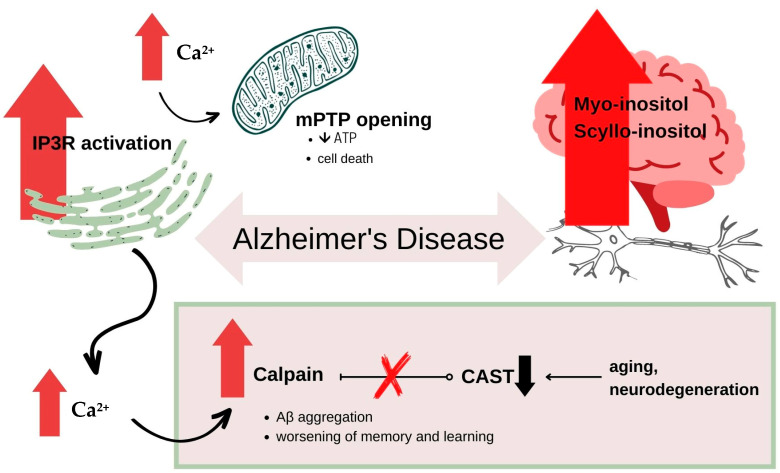
The summary of the mechanisms occurring in the course of AD in relation to inositol metabolism disorders. [ATP—adenosine triphosphate; mPTP—mitochondrial permeability transition pore; CAST—calpastatin; IP3R—inositol 1,4,5-triphosphate receptor].

In AD we can observe the unfavourable ratio of Aβ42 and Aβ40 levels, with a predominance of Aβ42—the more neurodegenerating one [46,47]. In AD patients according to the literature inositol metabolism is disturbed in several ways. It was proven that AD patients have higher concentrations of MI and SI in their brains compared to healthy people, despite of pathogenesis of the illness [48,49,50]. In Firbank et al. paper AD patients’ MI levels seemed to be raised by about 15% but with no evidence for an increase in any particular brain region. Higher MI levels are also seen in adults with Down’s syndrome and it has been shown that these patients will mostly develop AD in the future [51]. High MI levels are also detected in presymptomatic patients and those with mild cognitive impairment (MCI), which indicates the possibility of using MI as a marker of the early stages of AD [48,52,53,54]. The metabolism of inositol in AD patients can be impaired at every stage causing the deregulation of Ca^2+^ neuronal homeostasis which leads to neurodegenerative disorders. The intracellular calcium concentration must be constantly maintained at the appropriate level for all processes to take place properly. Disturbances in calcium levels can lead to serious malfunctions in its functioning. Calcium regulates the processes of growth, development and apoptosis, and its inadequate level can even lead to cell death [55,56,57,58]. In human cells, the level of calcium is regulated by several mechanisms. Calcium can enter cells from the extracellular space or it can be taken from the intracellular calcium store, which is the ER. Many studies indicate the enormous role of MI derivatives in regulating these processes. The role of IP3 in the elevation of intracellular calcium levels by releasing it from ER was described earlier in this work, but it is also worth mentioning that the activation of calcium influx from the extracellular space—the so-called Ca-influx also plays a significant role in the pathomechanism of AD and other neurodegenerative disorders [59,60]. There are reports indicating that Ca-influx can be as well stimulated by another MI derivative—Inositol 1,3,4,5-tetrakisphosphate (IP4) [61,62]. Many papers considering this topic focused on disruptions to the function of IP3R. Former literature report a decreased level of binding sites of IP3R in AD patients [63,64] and more recent studies highlight the over-activation of these channels. There is a strong correlation between the overactivity of IP3R and mutations in the presenilin genes [65,66,67]. In presenilin transgenic mice (PS1 FAD mutant mice) increased activity of IP3R was observed which subsequently enhances IICR and leads to increased intracellular calcium levels. Under conditions of increased Ca^2+^ levels, together with the additional oxidative stress characteristic of AD, a non-specific pore in the inner mitochondrial membrane is opened, known as the mitochondrial permeability transition pore (mPTP). The opening of mPTP allows the passage of molecules smaller than 1.5 kDa, and therefore, also protons. Consequently, this leads to the depletion of ATP stores and cell death [25,68,69,70]. It was proven that genetic modifications including the reduction of IP3R1 contributed to the equalization of Ca^2+^ levels [25,71]. Moreover, elevated levels of calcium activate calpain—a protein belonging to the family of calcium-dependent, non-lysosomal cystine proteases. Calpain has many isoforms, but to date, two of them have been most extensively studied—μ-calpain and m-calpain, otherwise known as calpain 1 and calpain 2, respectively. Both of them are activated by calcium ions and inhibited by a specific endogenous inhibitor—calpastatin (CAST). Calpain regulates many processes inside the cell, including the proper course of the cell cycle, cell proliferation, signal transduction, proper functioning of key protein kinases and phosphatases, apoptosis, memory, learning and in neurons—long term potentiation (LTP) which is a specific function for this type of cell [72,73]. With increasing age and in the course of neurodegenerative disorders such as AD, there is an excessive, uncontrolled activation of calpain, as a response to higher levels of intracellular Ca^2+^. It was shown that CAST concentrations are additionally lowered in the prefrontal cortex in AD patients, thus, apart from the over-stimulation of calpain by calcium, its inhibition also does not work properly [74,75]. Overly elevated calpain promotes the aggregation of Aβ and NFTs, as well as induces the dephosphorylation (deactivation) of cAMP-response element-binding protein (CREB)—a key protein essential for converting a short-term memory to a long-term memory and other memory and learning processes [76]. Opinions about the role of calcium in the process of direct cell damage are divided, and the authors now tend to emphasize the subtle effects of calcium on the slow progression of neurodegenerative changes, rather than the rapid process leading to cell death [29,77]. Another interesting linkage between disturbances in MI metabolism and the presence of AD is MI’s influence on catalase. Catalase allows the H_2_O_2_ to break down to harmless water and oxygen, and thus, together with other antioxidants such as glutathione peroxidase, reduces the formation of reactive oxygen species (ROS) and saves the cells from oxidative stress [78]. One of the most recent works on the influence of MI on the course of AD focuses on the inhibition of catalase function by MI, while other polyols such as mannitol, sorbitol and glycerol have been shown to have an activating effect on catalase. Researchers suggest that the inhibitory effect of MI may be due to MI binding to an active site of catalase or that MI causes conformational changes in the structure which can result in loss of activity. The loss of the antioxidant capacity of catalase may result in the occurrence of oxidative stress in cells, which was proven to be significantly increased in the course of AD and other neurodegenerative diseases [53,79]. On the other hand, previous reports suggest that MI supplementation results in an increase in the activity of antioxidant agents. Jiang et al. research has shown that antioxidative enzymes’ activity such as catalase, glutathione peroxidase and glutathione reductase were improved along with the increasing MI levels in the diet of juvenile Jian carp (*Cyprinus carpio* var. *Jian*) [80]. Information on processes related to disturbances in the biochemistry of inositols that lead to neurodegeneration in the course of AD has been posted in Figure 3.

### 6.2. Parkinson’s Disease

Parkinson’s Disease (PD) is the second most common neurodegenerative disease after AD, occurring in about 2–3% of people over the age of 65. In its course, there is a gradual loss of substantia nigra neurons leading to a decrease in dopamine levels in the striatal brain regions and the deposition of intracellular aggregates of α-synuclein. Many researchers also emphasize the important role of progressive dysfunction of mitochondrial function and the resulting increase in oxidative stress and increase in ROS. Clinical manifestations of PD include bradykinesia and at least one of the additional motor impairments: rigidity or rest tremor. Motor disorders usually occur asymmetrically [81].

Research on animals and cells has shown that IP6 has a protective effect on nerve cells in models of Parkinson’s disease. Researchers have demonstrated significant protection against ROS and lipid peroxidation product malondialdehyde (MDA) in PD’s model cells—6-OHDA (6-hydroxydopamine) previously exposed to IP6. IP6 was shown to inhibit the activation of key proteins involved in apoptosis. Therefore, IP6 due to its anti-apoptotic effect is proven to be neuroprotective against the loss of dopaminergic neurons in PD model cells [82,83].

Considering PD, the researchers also focused on examining the levels of receptors for IP3 and, as in older studies on AD, showed reduced levels of IP3 binding sites. A 50% reduced level of IP3 binding sites in the caudate nucleus, putamen and pallidum with no differences in the frontal cortex at the same time [84].

PD is still diagnosed based on the presence of characteristic clinical symptoms when the disease is already at a significant stage. Many authors have searched for potential diagnostic biomarkers of PD and other neurodegenerative diseases. Magnetic resonance spectroscopy (MRS) is a useful tool that allows for quantitative, non-invasive analysis of selected brain region biochemistry [85]. Of the substances present in the brain that have been studied, many researchers have focused on evaluating the concentration of MI. In Mazuel et al.’s research PD patients and healthy controls were examined with the use of hydrogen 1 proton magnetic resonance spectroscopy (^1^H-MRS) for the assessment of the metabolic profile in the putamen. As a result, the MI was significantly lower in drug-off PD patients and the MI level did not change even when the therapy was reintroduced [86]. Gröger et al. also used ^1^H-MRS to investigate the distribution of brain metabolites and focused on the ratios between different substations. It has been shown that the rostral substantia nigra regions of PD patients showed a trend towards decreased MI and total creatine (creatine + phosphocreatine, tCr) ratio compared with healthy controls whereas in the caudal substantia nigra regions the MI/Cr ratios were increased in PD patients compared with healthy controls [87,88].

Progressive Supranuclear Palsy (PSP) also known as Steele–Richardson–Olszewski syndrome is a chronic neurodegenerative disorder also involving the deposition of Tau protein aggregates, clinically resembling PD, and therefore, was often being misdiagnosed for this reason in the past. Typical symptoms of PSP include bradykinesia with disproportionate postural instability, upright posture with neck stiffness, frontal behavioral and cognitive changes, vertical gaze palsy and other brainstem deficits [89]. One of the areas of the brain affected by the disease is the Supplementary Motor Area (SMA). An Italian group of researchers focused on demonstrating PSP-specific neurobiochemical changes within the SMA using ^1^H-MRS [36]. Research has shown reduced scyllo-inositol levels in the SMA portion of the brains of PSP patients. The researchers stress that there are reports stating the SI’s neuroprotective ability against the deposition of protein aggregates in neurodegenerative diseases. In another paper, researchers administered 12 g of inositol daily to nine PD patients. They showed no significant positive MI effects but suggested possible beneficial effects of supplementation in patients with additional depressive symptoms and anxiety [90].

### 6.3. Huntington’s Disease

Huntington’s disease (HD) is a progressive, autosomal-dominant neurodegenerative disorder, manifested by chorea, dystonia, decreased motor coordination, cognitive decline and behavioral changes. Usually, the first symptoms of the disease appear in middle age but may occur at any stage of the patient’s life [91,92]. The genetic basis of HD is an occurrence of expanded CAG repeat in exon 1 of the huntingtin gene (polyglutamine, polyQ expansion) causing the formation of mutant huntingtin (mHtt) protein. mHtt occurs is found in all types of cells, but it appears to be toxic only for neurons of specific brain regions: cortex, caudate nucleus and putamen [25]. Many reports confirm, that in HD deregulation of IP3R also takes place, this time due to the direct binding of mHtt protein to IP3R1 in its C-terminal region [29]. It was proven that mHtt sensitized activation of IP3R by IP3 in medium spiny striatal neurons (MSNs), while normal huntingtin protein showed no such ability. HD transgenic model mice used in research considering alterations in IP3R functions are yeast artificial chromosome (YAC) and mice with a targeted disruption of both Htt-associated protein-1A (HAP1) gene alleles. HAP1 was the first Htt-binding protein to be found. It was shown that HAP1 is present in MSNs and it intensifies the action of mHtt, resulting in increased IICR and elevated calcium levels [93]. Moreover, IP3R is an allosteric protein and its function can be disrupted by changes in its conformation. One of the enzymes that can disturb IP3R’s allostery is transglutaminase type 2 (TG2) and its regulation is based on the covalent posttranslational modification of the Gln2746 residue which TG2 tethers to the communicating subunit [94]. Both Gln2746 changes and IP3R dysregulation were detected in HD models, confirming the involvement of IP3R dysregulation in disease pathogenesis [29,95]. TG2 ablation in model mice prolonged their lifespan and improved motor function [25]. Other researchers report that in the course of HD, there is an increase in the activity of inositol hexakisphosphate kinase type 2 (IP6K2), which, among other things, leads to a reduction in Akt phosphorylation, which may lead to cell death and promotes autophagy. Upon activation, IP6K2 is released from the nucleoplasm into the cytoplasm. It was shown that in HD lymphoblasts IP6K2 was present mainly in the cytoplasm of the cell, while in control cells it remained in the nucleus. IP6K2 catalyzes the reaction of transforming IP6 into inositol pyrophosphate diphosphoinositolpentakisphopshate (IP7; PPIP5). In HD lymphoblast cells, increased levels of IP7 were detected compared to control cells, and IP7 promotes cell death [96,97].

### 6.4. Spinocerebellar Ataxias

Another neurodegenerative disorder group that will be discussed in this paper is Spinocerebellar Ataxias (SCAs). SCAs are a group of hereditary autosomal dominant neurodegenerative disorders characterized by progressive ataxia. To date, there are more than 40 identified types of SCA [98]. Each of the SCA types are based on a mutation, deletion or polyglutamine (poly-Q) expansion at a different gene locus [99]. The characteristic symptoms in the course of SCA include loss of balance and coordination, dysarthria and nystagmus [100]. SCAs cause damage to the brain tissue and mostly affects the cerebellar Purkinje neurons, leading to cerebellar cortex atrophy, neuronal loss, and irreversible degeneration of spinocerebellar tracts, dentate nucleus and pontine[101]. There are many reports in the literature on the relationship between SCAs and dysregulation of IP3R function. Some papers indicate that reduced IP3R abundance is observed in SCAs, while others emphasize the phenomenon of hypersensitivity of IP3R for its ligand—IP3, and excessive IP3R activation [102].

SCA1, SCA2 and SCA3 are caused by intracellular poly-Q (CAG) expansions of cytosolic ataxin protein. It is already known that researchers are looking for potential diagnostic biomarkers for the effective detection of neurodegenerative diseases. In the Oz et al. paper, researchers focused on the analysis of levels of total NAA (*N*-acetylaspartate + *N*-acetylaspartylglutamate, tNAA), glutamate, glutamine, tCr and MI. The levels of tNAA and glutamate were decreased, while glutamine, tCR and MI levels were elevated. Along with increased neuronal loss and astrocytosis, elevated levels of MI were detected in the pons and cerebellar hemispheres of SCA 1 patients. Differences in MI levels in the study groups allowed for the complete separation of patient and healthy controls (104). In SCA2 there is the occurrence of Purkinje cells depletion and degeneration of the inferior olives, pontine nuclei, as well as pontocerebellar fibers. Ataxin protein-2 specifically binds the IP3R and increases IP3R sensitivity to its activation by IP3. Due to the IP3R overactivation supranormal calcium release from Purkinje cells’ endoplasmic reticulum can be observed and as mentioned above in this paper, elevated levels of Ca^2+^ play a key role in the development of neurodegenerative pathologies. In the paper by Kasumu et al., the authors present a study on a SCA2-58Q transgenic mouse model. In SCA2-58Q mice the IP3R activation is chronically downregulated consequently lowering related calcium signaling. This downregulation is obtained through overexpression of the Inositol 1,4,5-phosphatase enzyme (5PP) in Purkinje cells of SCA2 transgenic mice. Their results supported the hypothesis that 5PP overexpression suppresses InsP3-induced Ca^2+^ release, and moreover, that Ca^2+^ release suppression prevents the progressive dysfunction of Purkinje cells in SCA2 mice [99]. SCA-3 is less severe than SCA-2 (25).

The deletion of the IP3R1 gene located on chromosome 3 leads to SCA type 15/16 with late/adult onset, SCA 29 with an early onset type, and also sporadic infantile-onset SCA [103,104,105,106]. Researchers suggest that deletion of the IP3R/ITPR1 gene leads to impaired IP3R function. A Western blot of lymphoblastoid cell line proteins confirmed reduced IP3R levels. In conclusion, gene mutations in the course of SCAs lead to reduced activity, as well as IP3R levels.

### 6.5. Other Neurodegenerative Disorders

The summary of other neurodegenerative disorders has been described in Table 2.

## 7. Possible Treatment Ideas

Changes in the level and metabolism of MI have long been attempted to relate to the occurrence of neurodegenerative diseases, and since these assumptions have been confirmed by many researchers, the next natural step has been to look for possible treatment ideas. From searching the research databases, much information on possible substances that affect the regulation of inositol metabolism can be found, some of them have already been tried, while others are only a vision of the future, knowing only the points of their grip in the cells, giving researchers the opportunity to discover them.

### 7.1. MI Itself

The first substance worth mentioning is the use of MI itself. MI is often administered in the form of d-pinitol (3-Omethyl-d-chiro-inositol), which is abundant in many plant families such as Pinaceae, Asteraceae, Caryophyllaceae, Zygophyllaceous, Cupressaceae, Aristolochiaceae and Sapindaceae [114]. In other diseases where MI levels are reduced, such as diabetes, PCOS, cancer and mental disorders: depression, panic disorder, obsessive-compulsive disorder and schizophrenia, inositol supplementation as a form of adjunctive therapy has already been better studied [21,115]. d-pinitol can be used in patients after stroke or epileptic seizures, where the loss of neuronal function and, at the same time, a decreased concentration of MI can be observed. In a study from 1996 in which inositol was administered to AD patients, there was a slight improvement in their condition, but these results were not statistically significant [116]. Other studies suggest that inositol may be a possible therapeutic agent of AD because of its positive effect on lowering the toxicity of Aβ oligomers due to the activation of insulin/IGF signaling components and at the same time regulation of γ-secretase activity. d-pinitol also has anti-inflammatory effects [115]. Currently, there are increasingly high hopes for the use of d-pinitol as an ameliorative agent in the treatment of AD. It is considered a selective γ-secretase modulator, and therefore, potentially it would not interfere with other enzymatic pathways, and could have a beneficial effect by inhibiting Aβ production. d-pinitol (NIC5-15) clinical trials are still unfinished, but this substance has a high potential for alleviating AD symptoms [114]. Insulin resistance contributes to the occurrence and worsening of symptoms of neurodegenerative diseases, so d-pinitol as an insulin sensitizer can play an important role in the inhibition of brain alterations. Medina-Vera et al. have shown that oral supplementation of d-pinitol lowered tau phosphorylation, by regulating cyclin-dependent kinase 5 (CDK-5) activity [117]. Scyllo-inositol, as mentioned above, is another naturally occurring stereoisomer of MI and it is also considered a potential remedy for neurodegenerative diseases. It was proven that SI is able to stabilize Aβ42, neutralize cell-derived Aβ trimers, promote low molecular weight Aβ in vivo and inhibit the aggregation of α-synuclein in Parkinson’s disease [30,32,82]. SI treatment results in a higher level of MI in the brain and cerebrospinal fluid (CSF). Furthermore, it was shown that these elevated levels of SI have no effect on phosphatidylinositol lipid production and SI does not incorporate into brain PI lipids; therefore, it does not affect the proper functioning of the PI lipids dependent signal transduction pathways. What is more, SI can be supplemented in a convenient oral form, with a high ability to penetrate the blood-brain barrier [118]. There was scientific research on transgenic mice (TgCRND8 mouse model of AD) supplemented by SI for two months, starting at 5 months of age. Mice consumed about 25–30 mg of SI daily. The treatment resulted in a significant reduction in insoluble Aβ40 and Aβ42 levels in the brain, in comparison to the healthy controls. The SI supplementation also reduced the percentage of the brain area covered with plaques [119]. There were seven clinical trials for scyllo-inositol (drug name: ELND005). Four of them were completed, while two were terminated and one is currently under recruitment. One of the completed trials was a randomized, double-blind, placebo-controlled clinical trial of SI conducted on patients with Down Syndrome (DS). DS patients have a high risk of developing AD with an early onset, because of the constant overproduction of Aβ. In this clinical trial, 23 patients were dived into four groups: placebo and three ELND005 administered orally groups with different doses (250 mg, 1000 mg, 2000 mg). The last two groups with the highest SI doses were withdrawn, due to a higher risk of respiratory infections. Patients consuming 250 mg of SI were divided into two groups, considering the number of daily doses: BID—received 250 mg twice a day and QD—once a day. Both groups—250 mg and placebo were observed for 4 weeks. This study confirmed that the administration of SI was well tolerated by patients and no newly identified safety risks were observed. Another trial on 353 patients with the exact same doses, but 78 weeks observation time, also confirmed a higher risk of respiratory tract infections in the group consuming 1000 and 2000 mg of SI, which also lead to more frequent withdrawals in this group connected with adverse events [120,121]. Ways of producing SI through metabolic engineering are currently being sought. In one of the latest studies on this subject, researchers report the possibility of using the bacterium—Corynebacterium glutamicum as a promising host for the production of SI [122]. Another study also aimed at finding inexpensive ways of SI production on a larger scale has shown that different bacteria—Bacillus subtilis can also be used as a potential cell factory [123]. Another MI stereoisomer—epi-inositol—has the ability to stabilize small aggregates of Aβ42, which lowers the Aβ induced toxicity in Alzheimer’s disease. It was proven that scyllo-insitol has the same effect on stabilizing Aβ [30,118].

### 7.2. Lithium and Valproic Acid

Lithium is currently the most widely used first-line mood stabilizer in the treatment of bipolar disorder. There are more and more reports about the possibility of using lithium also in neurodegenerative diseases. Lithium has many mechanisms of action, but the neuroprotective effects can be obtained due to its influence on inositol metabolism by inhibiting IMPase, causing the decrease in MI levels and consequently lowering the IP3 levels. Lithium-induced autophagy leads to the clearance of neurodegenerative disease-related proteins such as mutant huntingtin fragments and mutant alfa-synuclein [124,125]. As lithium lowers MI levels, it has also been studied how the drug will act with an additional reduction in dietary MI during its use. The results of the research showed an increase in the effect of lithium when following a diet reduced in inositol. The administration of lithium alone reduced the concentration of brain MI by 4.7%, while combined with a low-MI diet by as much as 10.8% [125]. Other mechanisms of lithium action include the regulation of apoptosis and the down-regulation of calpain. The authors emphasize that further clinical studies are needed to determine, whether this drug can be used in the treatment of neurodegenerative diseases and that therapy based only on lithium should not be considered due to the inconsistent efficacy and the occurrence of potential side effects. Lithium should be considered as an adjuvant in the treatment of neurodegenerative diseases, administered along with other existing therapeutic methods [126]. Lithium may also be helpful in the treatment of spinocerebellar ataxias, thus it can alleviate the cerebellar symptoms in affected patients [127]. There are also clinical studies on specific IMPase inhibitor (L-690,330) that mimics the effects of lithium in reducing the MI levels which leads to inhibition of the phosphoinositide cycle, consequently inducing autophagy and clearance of mutant proteins occurring in HD, PD and other neurodegenerative disorders [128]. Another mood stabilizer also a candidate drug for the treatment of neurodegenerative diseases is valproic acid (VPA). Both VPA and other anticonvulsants, such as carbamazepine, also reduce the intracellular concentration of inositol in the brain, having the same effect as lithium and specific IMPase inhibitors. VPA, on the other hand, acts by inhibiting MIPS, which catalyzes the reaction preceding the formation of free MI conducted by IMPase, consequently leading to the same effect of promoting autophagy and mutant protein clearance [112,124]. Other authors also report that during pharmacotherapy with valproic acid and lithium, along with clinical improvement of the patient’s health, there is a visible reduction in MI‘s biosynthesis and its concentration in brain regions such as the frontal and temporal lobes, basal ganglia and cingulate of the rim [3]. Many studies on mice models and rats have shown that lithium and VPA decrease the Aβ plaque formation, enhance Aβ degradation, reduce tau phosphorylation and generally improve neurodegenerative processes [128].

### 7.3. γ-Secretase Inhibitors

γ-secretase is one of three secretases participating in the cleavage of APP and it catalyzes the last reaction when the Aβ is formed. Under normal conditions, only small amounts of Aβ are produced in cells, but in cases of mutated PS 1,2 and APP, these proteins are more easily accessible for γ-secretase, which leads to uncontrolled overproduction of Aβ Attempts have been made to suppress the action of γ-secretase through its inhibitors and modulators. Modulator function was based on the increasing formation of soluble short-chain Aβ, creating a favorable ratio (Aβ37:Aβ40) and reducing the formation of Aβ42. Unfortunately, more than half of the clinical trials of γ-secretase inhibitors have not been finished, due to complications including limited effectiveness, large side effects and poor pharmacokinetics [129,130].

### 7.4. Other Possibilities

Additionally, some newly identified autoantibodies were described by Greek researchers and correlated with cerebellar disorders. In Purkinje neurons the antibody against receptor 1 for inositol was found. That autoantibody can be pivotal in seizures in mice with congenital defects for ataxy and epilepsy. That also gives a chance to develop immunotherapy in such disorders [131].

There are also reports about 2-aminoethoxy diphenyl borate—membrane-permeant IP3R blocker. The authors suggest its neuroprotective function through the inhibition of IP3R-related calcium release. The usage of 2-aminoethoxy diphenyl borate seems promising due to its therapeutic potential for the treatment of neurodegenerative disorders [25,132,133]. The summary of possible therapeutic methods found in literature is presented in Table 3.

## 8. Conclusions

In summary, the incidence of neurodegenerative diseases in society is still a significant problem, affecting mainly the elderly. The researchers are still looking for effective methods of detecting neurodegenerative diseases at their early stages, for which the level of MI can prove to be useful. Changes in MI metabolism at various stages can contribute to the development of diseases leading to atrophy of brain structures, either by promoting the deposition of mutant proteins or an enhancement of calcium release, which ultimately results in increased neuronal degeneration. The use of appropriate supplementation, which would significantly increase the quality of life in affected patients is highly desirable. Studies on the therapeutic efficacy of MI, d-pinitol, SI and other possible treatments should still be conducted. However, the use of MI appears to be beneficial, and supplementation with MI in appropriate doses may prove effective in patients with neurodegenerative diseases with associated symptoms of depression and anxiety.

## Figures and Tables

**Table 1:** Structural formulas of primary cyclitols.

Cyclitol 1,2,3,4-cyclohexenetetrol	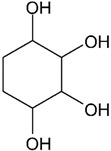
Myo-inositol	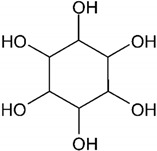
Pinitol	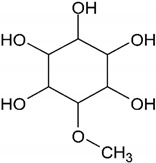

**Table 2:** The summary of other neurodegenerative disorders.

Disorder	Linkage to MI and It’s Derivatives	References
Amyotrophic Lateral Sclerosis (ALS)	- increased MI in ALS patients brains - concentrations of MI were correlated with disease severity	[107]
	- inositol hexakisphosphate kinase 2 promotes cell death of anterior horn cells in the spinal cord	[108]
	- total *N*-acetylaspartate to myo-inositol ratio (tNAA/mIns) in motor cortex is significantly declined - motor cortex tNAA/mIns ratio shows promise as a potential ALS biomarker	[109,110]
Niemann-Pick Disease type C (NPC)	- genetic alterations in lysosomal cholesterol transporter NPC1 disrupt expression and distribution of IP3R1 in ER of neurons thus leading to elevated calcium levels in cells causing mitochondrial cytotoxicity and cell death	[111]
Epilepsy	- regions of brain, involved in seizures, have higher level of inositol - suggested that inositol can induce epilepsy	[112]
	- elevated levels of MI in hippocampus - suggested that MI can be used as early predicator of epilepsy	[113]

**Table 3:** The summary of possible therapeutic methods found in literature.

Substance (Drug Name)	Function	Results	Diseases	References
d-pinitol	Insulin-sensitizer; Inhibits γ-secretase; Lowers Tau phosphorylation	↓ Aβ production	AD, tauopathies	[114,117,134,135]
Scyllo-inositol (ELND005, AZD-103)	Stabilize Aβ42, neutralize cell derived Aβ trimers, promote low molecular weight Aβ in vivo; Inhibits the aggregation of α-synuclein in Parkinson’s disease	Decreased neuronal toxicity, increased long-term potentiation (LTP) and ablation of cognitive deficits in multiple mouse models of AD	AD, PD	[30,120,122,123,136,137]
Epi-inositol	Stabilize Aβ42	Decreased aggregation of Aβ; Lowers anxiety	AD	[30,138]
Lithium; IMP-ase inhibitor (L-690,330)	Inhibits IMP-ase,	↓ MI ↓ IP3 ↑ clearance of autophagy substrates	HD, PD, SCAs	[112,124]
Valproic acid	Inhibits MIPS in human brain, lowers MI level, inhibits y-secretase	inhibits Aβ production	AD	[112]
γ-secretase inhibitors (Semagacestat (LY450139), MK 0752, E2012, GSI 136)	Inhibits γ-secretase and lowers β-amyloid in blood and spinal fluid in humans	Semagacestat did not slow disease progression, worsened cognitive functions in patients, study drug was stopped in all studies.	AD	[139,140]
Antibodies	Antibodies against IP3 receptor 1	This antibody may have a direct involvement in neurodegenerative process or can be a marker or cerebellar injury.	Cerebellar ataxia, epilepsy	[131]

## Data Availability

Not applicable.

## Abbreviations

^1^H-MRS	Hydrogen 1 proton magnetic resonance spectroscopy
5PP	Inositol 1,4,5-phosphatase enzyme
6-OHDA	6-hydroxydopamine
AD	Alzheimer’s disease
AICD	APP intracellular fragment
APP	Amyloid precursor protein
ATP	Adenosine triphosphate
Aβ	Amyloid β
CAST	Calpastatin
CDP-DAG	Cytidine diphosphate diacylglycerol
CFT	APP C-terminal fragment
CICR	Calcium induced calcium release
CMP	Cytidine Monophosphate
CSF	Cerebrospinal fluid
DAG	Diacylglycerol
ER	Endoplasmic reticulum
G-6-P	Glucose-6-Phosphate
GPI	Glycosyl-phosphatidyloinositol
GSIs	γ-secretase inhibitors
HAP1	Htt-associated protein-1A
HD	Huntington’s disease
HK	Hexokinase
Htt	Huntingtin
IICR	Inositol induced Ca-release
IMPase	Inositol monophosphatase-1
IP	Inositol phosphate
IP3	Inositol 1,4,5-triphosphate
IP3R	Inositol 1,4,5-triphosphate receptor
IP6K2	Hexakisphosphate kinase type 2
IPG	Inositiol-phosphoglycan
LTP	Long term potentiation
MCI	Mild cognitive impairment
MDA	Malondialdehyde
mHtt	Mutant huntingtin
MI	Myo-inositol
MIOX	Myo-inositol Oxygenase
MIPS	MI-phosphate synthase
mPTP	Mitochondrial permeability transition pore
MRS	Magnetic resonance spectroscopy
MSNs	medium spiny striatal neurons
NADPH	Nicotinamide adenine dinucleotide
NFTs	Neurofibrillary tangles
PD	Parkinson’s disease
PI/PtdIns	Phosphatidylinositol
PI4K	Phosphatidylinositol 4-kinase
PIP	Phosphatidyloinositide
PIP2	Phosphatidylinositol biphosphate
PIP5K1	Phosphatidylinositol phosphate 5-kinase 1
PIS	Phosphatidylinositol synthase
PLC	Phospholipase C
PPIP5	inositol pyrophosphate diphosphoinositolpentakisphopshate
PS 1,2	Presenilin 1,2
PSP	Progressive supranuclear palsy
ROS	Reactive oxygen species
RyR	Ryanodine receptor
SCA	Spinocerebellar ataxia
SMA	Supplementary Motor Area
tCr	Total creatine, creatine + phosphocreatine
TG2	Transglutaminase type 2
tNAA	Total NAA, *N*-acetylaspartate + *N*-acetylaspartylglutamate
VPA	Valproic acid
YAC	Yeast artificial chromosome
